# Preparing biomedical students for the unknown: Some unusual challenges for students to help them understand the fundamentals of empirical research

**DOI:** 10.15252/embr.201949004

**Published:** 2019-09-15

**Authors:** Adolfo Rivero‐Müller, Matthias Nees

**Affiliations:** ^1^ Department of Biochemistry and Molecular Biology Medical University of Lublin Lublin Poland; ^2^ Faculty of Science and Engineering, Cell Biology Åbo Akademi University Turku Finland; ^3^ Institute of Biomedicine University of Turku Turku Finland

**Keywords:** S&S: History & Philosophy of Science, Methods & Resources

## Abstract

Make teaching challenging again: a pedagogic approach to educate students on how to do science rather than learn about it.

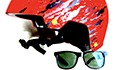

Curricula in the biomedical sciences are largely based on memorising knowledge along with accepted “archetypes” to describe biological systems, such as the central dogma of molecular biology. Establishing these profound insights in the 1960s and 1970s has been a true intellectual adventure for the scientists involved—but it is no more for students in the 21^st^ century. In fact, most undergraduate students memorise factual knowledge with little or no explanation of the creative approaches and ideas that have led to these breakthroughs and insights. Moreover, students are rarely trained on how to formulate a hypothesis, how to design conclusive experiments along with essential controls and how to conduct rigorous empirical science. Textbooks are no help either as most books contain—if at all—only small sections on developing a hypothesis, experimental design and methods. They also rarely highlight the rationale behind creative and ground‐breaking experiments, despite that such information would make exciting educational and even entertaining material.

… students are rarely trained on how to formulate a hypothesis, how to design conclusive experiments along with essential controls and how to conduct rigorous empirical science.

Such detailed descriptions of methods and technologies are only found in rather dry publications 1 that focus on methods, and that are written for experts in the field, not for students. As the result of novelty‐based publishing strategies, original publications contain little or no information on the trials and errors, frustrations, nor the “eureka” moments that researchers have experienced to make the experiment work. What is also missing are the group efforts and creative teamwork, without which biomedical research is unattainable. In addition, teaching science at universities does not systematically utilise the potential of interesting and entertaining narratives for educational and pedagogic purposes. It is now commonly accepted and proven that teaching “just the facts” is not an effective method 2.

Our goal is therefore to recreate the experience of failure and excitement that is typical for research, by posing seemingly impossible challenges that require all the elements that a scientist needs: inspiration, creativity, teamwork and thinking outside the box. This somewhat reminds us of the famous science fiction writer Arthur C. Clarke, and his three laws of technology. His 1^st^ law claims: “When a distinguished but elderly scientist states that something is possible, he is almost certainly right. When he states that something is impossible, he is very probably wrong”. Younger generation scientists need to keep this in mind. It is them, who will most likely discover new, revolutionary things—they just have to be prepared for it.

## Methods in teaching

In teaching, methods are most often presented as a necessary means to answer a particular biological question, but not as the fundamental basis for discovery. The description of technologies and methods is typically considered secondary to biological knowledge, which is conceived as the highest value. Even the best teaching units do not necessarily invite students to use creative thinking processes, motivate their inspiration and ignite their fascination to solve difficult problems.

Without an education in solving problems, students will have little opportunity to acquire this essential knowledge before they set a foot in the lab.

University lectures in natural sciences rarely highlight the multiple possibilities, alternative methods, variety of tools and their respective pros and cons. Method sections in scientific publications usually only provide the most essential information, often just referring to another publication, which may refer to yet older papers. Alternatively, methods are simply described as “performed using standard methods”, or “according to manufacturers’ instructions”. In other cases, a rudimentary technical paragraph, shortened beyond recognition, is placed somewhere in the supplementary material. As a result, students, once they start working in a research laboratory, often use techniques without fully understanding them, and ready‐to‐use kits containing solutions A, B and C only persevere this situation. There is an urgent need to bring methods, techniques and the experimental design back to the attention of teaching.

The excitement for the fantastic possibilities of omics technologies often lasts only to the point when students need to make sense of exceedingly complex data sets.

With the rise of social media, an increasing number of online lectures are now offered by virtual institutes, such as Khan Academy (https://www.khanacademy.org/), Open University (http://www.open.ac.uk) or an offshoot of MIT (https://ocw.mit.edu/index.htm). In principle, everyone can attend these courses at their own pace. Most of this information is simply meant to be “consumed”, and much of the material is not questioned or even understood at its roots. Unfortunately, this does not prepare students properly for real‐life situations, in which researchers *ad nauseam* face unprecedented situations, unexplained failures and complex results that defy interpretation. Without an education in solving problems, students will have little opportunity to acquire this essential knowledge before they set a foot in the laboratory.

## The ultracool method

Many undergraduate students think that a single “cool” technique can solve almost all problems. A good example is next‐generation sequencing (NGS) that promises to give a detailed, almost magical, snapshot of what cells or tissues are doing at any particular moment or situation. But it misses all the changes that occur at the protein and metabolic level and the myriad of interactions between metabolism, proteins and nucleic acids. We do not object to the use of NGS, of course. Nevertheless, it is very difficult to see the forest for the trees in thousands of transcripts. True understanding requires additional data, such as the turnover and stability of mRNAs, alternative splicing or the efficacy with which they are translated into proteins, and what is the functional half‐life of these proteins. Without bioinformatics, statistics and suitable software tools, this deluge of information may be even counterproductive and misleading. NGS also does not provide an understanding of the pronounced heterogeneity and dynamics in tissues, cells and cell lines, unless single‐cell sequencing is used. The same applies to proteomics and metabolomics, technologies that equally generate an overflow of data. The excitement for the fantastic possibilities of omics technologies often lasts only to the point when students need to make sense of exceedingly complex data sets.

Another example for the disconnect between data generation and knowledge are genome‐wide association studies (GWAS). These deliver a spectrum of different correlations between single nucleotide polymorphisms (SNPs), and a disease or specific traits such as body height. One of the key problems with GWAS is that these variations only account for a disappointingly small amount of cases and phenotypical manifestations. A recent meta‐study showed that as many as 100,000 SNPs in the human genome may influence various phenotypes, yet the individual effect of single SNPs is only miniscule. Even the cumulative effect of all variations combined does not fully explain a particular phenotype 3. It is not our goal to state that GWAS are generally useless or treacherous. We rather try to point out that young researchers should develop a critical mind, given the complexity of biology. We enjoy showing students clear but misleading associations such as between ice‐cream sales and shark attacks (https://www.ibpsychmatters.com/why-correlation-is-not-causation) and other examples (http://www.tylervigen.com/spurious-correlations) to prove our point.

## Prepare for the unknown

How should we teach future researchers to effectively prepare for the unknown? In the laboratory, students have to deal with failure 4, stress and frustration on their own. In a classroom, however, we can build upon failure, help them to grow and think critically. In the following sections, we describe what we consider as a possible scenario for how undergraduate students in the biomedical sciences could be trained. We have named our approach “challenges”: difficult problems that students need be solved in a safe space that allows them to deal with failures, lack of ideas and misconceptions. This approach exceeds and expands the very basic concept of “problem‐based learning”. Our very special challenges are often exaggerated and of fake “global relevance”—whilst simultaneously having a strong entertaining quality that helps dealing with pressure.

What is a good challenge? In our view, challenges should take the students out of their comfort zone; be based on an unheard of and untraceable problem; have no obvious answer; and have potentially multiple answers and multiple steps. Interesting challenges should trigger independent thinking: the problems should be exciting and strange, but serious enough to relate at least in principle to real‐life situations. They could be classified as “fake problems” with a very realistic touch.

The alien is the prototype of the unknown, the unfamiliar, the ultimate mystery.

What better analogy to our approach than the behaviour of adult guillemots, a.k.a. murres (*Uria aalge* and *Uria lomvia*), who “encourage” their chicks to jump off the nesting cliff before their wings are fully developed. The poor chicks frenetically flap their stunted wings in a desperate attempt to overcome gravity and gain enough distance from the sharp boulders at the bottom. Despite what seems evolutionarily counterintuitive, these chicks grow much faster 5. Taking the big plunge might also be the best way to encourage students to fly on their own. In traditional teaching, this rarely ever happens.

## Out of this world

The first two points on our list of ideal components for challenges can be tackled simultaneously, by using “out of this world” topics—literally. For this purpose, we typically pick extra‐terrestrials, mythological, extinct species and imaginary creatures as subjects—from here onwards all are referred to as “aliens”. The alien is the prototype of the unknown, the unfamiliar, the ultimate mystery. It has a potentially threatening connotation and stands for demanding situations that are notoriously difficult to solve, and for which, our current knowledge may be insufficient. This is partly modelled on real‐life situations such as outbreaks like Ebola or Zika virus. The use of alien biology further allows us to let our imagination fly. Aliens are usually dangerous, evil and selfish. The closest to this concept we know of is an online game, developed by the Genetic Science Learning Center (http://learn.genetics.utah.edu/content/genetherapy/doctor/), found by one of our students—which demonstrates how skilful students are in mining information from the Internet. They are indeed such excellent data miners to the degree that it deters them from thinking independently.

In biology, organisms with truly alien properties are abundant, and the deeper we look the weirder they seem.

Students are surprised when we present the webpage of—in our case, Finnish and Polish—immigration offices, where terms such as *alien registry* and *the rights of aliens* are described. As good legal aliens, both authors registered with their 6 slimy tentacles and 5 pairs of eyes in those offices. In biology, organisms with truly alien properties are abundant, and the deeper we look the weirder they seem. By way of example, it was recently found that chloroplasts of the marine green algae *Boodlea composita* contain single‐stranded DNA (ssDNA) genomes 6. This abnormity prompted us to change the slides in our molecular biology course, where DNA was always double‐stranded (dsDNA) in living organisms. Thus, not even dogmas show consistency in biology. In fact, the chloroplast genome of *B. composita* is largely composed of palindromic sequences that form multiple dsDNA hairpins, practically being quasi‐dsDNA.

Another great source of preposterous problems comes from parasites, especially viruses. There are literally no boundaries for the ingenuity of viral replication cycles, genome structure and organisation, or their interactions with host cells. Viruses can carry almost any type of genetic material, including ssDNA, dsRNA and ssRNA. Viruses have also challenged the central dogma of molecular biology by inventing reverse transcription (RT). Parasites also utilise a broad spectrum of alien or abnormal behaviours. Some parasites use fascinating life cycles that make Ridley Scott's Xenomorph—commonly referred to as “the Alien”—look almost like a simpleton. In fact, “the Alien” might have been inspired by the complex life cycle of parasitic crustaceans such as *Phronima* sp. (http://theconversation.com/meet-phronima-the-barrel-riding-parasite-that-inspired-the-movie-alien-22555).

Moreover, there are plenty of mind‐blowing examples of how parasites can control and manipulate their hosts. For example, fungi of the genus *Cordyceps* grow inside insects’ bodies and effectively alter the neuromuscular levels of control, turning their victims into “zombie ants”. The mind‐controlled insects are compelled to climb to the uppermost tip of a branch, high above the colony and clamp their jaws, until they die. In this exposed position, the fruiting body of the fungus eventually erupts from the insects’ body, spreading spores that may infect many additional insects, with the potential to wipe out entire colonies. Likewise, less than a decade ago, nobody would have thought that our gut bacteria are able to control our emotions, but mounting evidence shows otherwise. Successful parasites can also exploit incredibly complex molecular systems to control their hosts. This can be illustrated by transcription activator‐like effectors (TALEs), fascinating modular proteins secreted by numerous species of *Xanthomonas*, plant parasitic bacteria. TALEs have the potential to modulate gene expression in plant cells with the goal to facilitate colonisation and survival for the parasite 7. Taken together, parasites, symbionts and marvellously odd creatures are extremely fruitful subjects to generate “educational challenges”.

## “Unheard of” or untraceable problems

**Figure 1:**
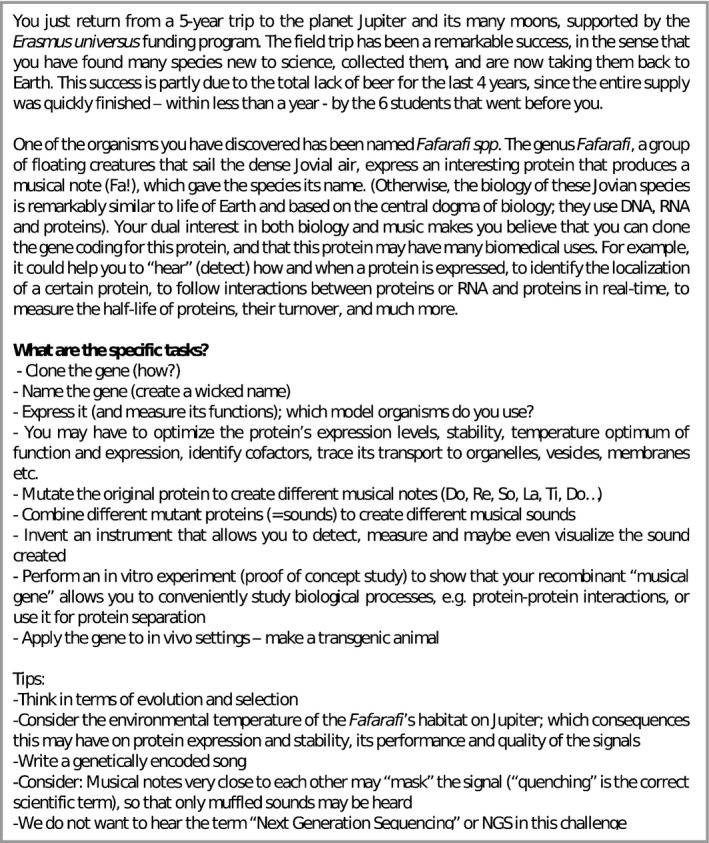
Challenge 1

**Figure 2:**
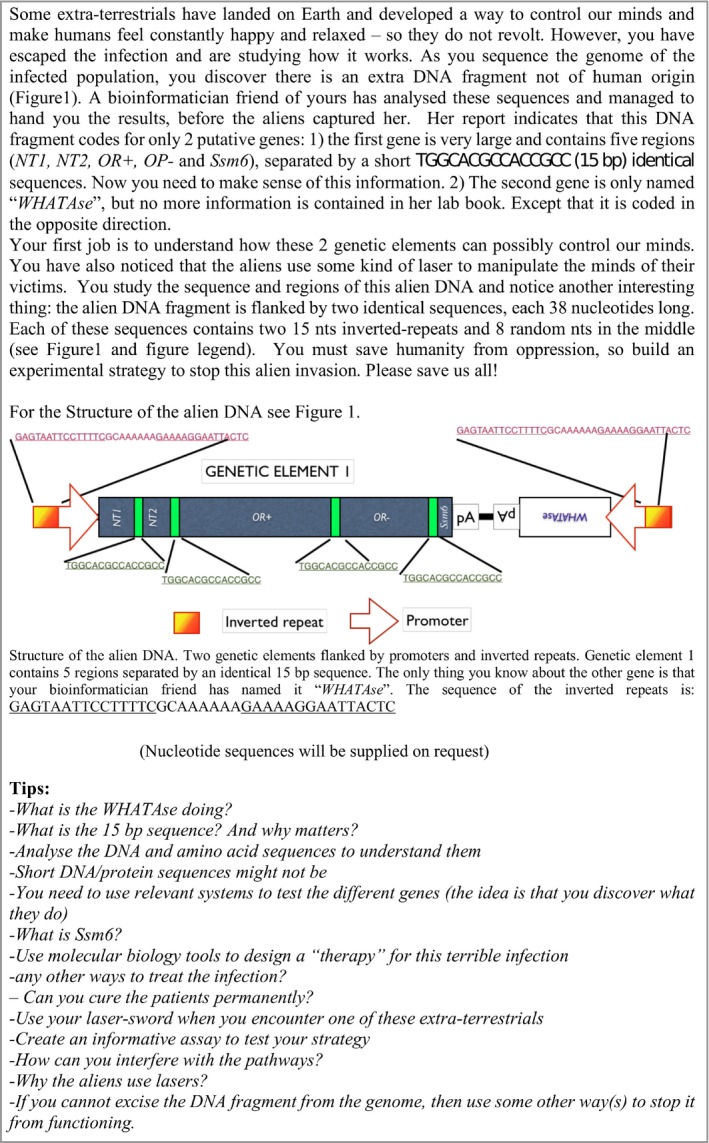
Challenge 2

The unheard of/untraceable problem requires enough information for solving a challenge but does not match to results by search engines. Such riddles need to compel students to use their imagination and creativity, rather than conveniently search the Internet for a solution. One way to create such “impossible” challenges is by switching physical properties, turning colour into sound (see challenge 1, Fig 1), or by exchanging nucleotide to amino acid sequences (see challenge 2, Fig 2). Our challenges are perfectly captured by the 2^nd^ law of Arthur C Clarke: “The only way of discovering the limits of the possible is to venture a little way past them into the impossible”.

A challenge is based on the thorough study of a biological phenomenon and it requires creating tools to study the alien creatures, and measure properties or phenotypes.

In many challenges, we include the need to create specific equipment to measure critical physical parameters. This is an additional challenge for biomedical students, who have little experience with engineering. The central idea here is that students need to devise simple, but reliable and effective ways to measure different phenomena and properties. It also prompts them to move into unknown and fantastical territory. Maybe this is captured by the 3^rd^ law of Arthur C Clarke: “Any sufficiently advanced technology is indistinguishable from magic”.

## No obvious answer

Probably, the worst possible way to test students is the use of standardised questions. In the biomedical sciences, this is commonplace, as memorising facts (not primarily understanding) is rewarded. In a typical challenge, on the other hand, we try to avoid excessive memorising and focus on understanding and promoting creativity instead. A challenge is based on the thorough study of a biological phenomenon, and it requires creating tools to study the alien creatures, and measure properties or phenotypes. This is followed by a test phase (developing bioassays and proof of concept studies), in which both are combined. Only then, is it possible to solve the big picture—or save humanity from extinction. Often, we add deliberate diversions, e.g. alternatives to choose from, different things one can do using the same tools, or options for diverse properties to be analysed. Most of our challenges are based on the discovery of specific genes or functional proteins. To add relevance, these functions typically represent a link between earthly and alien biology.

The challenges should comprise one or several problems, and many possible ways exist to an acceptable solution. For example, we may start by cloning a gene by traditional restriction–ligation or more modern cloning strategies such as LIC, gateway or recombineering. Students should justify their choice and briefly outline the advantages and limitations of these techniques. They also need to specify why a certain technique was selected versus others. The teachers may encourage, or narrow down, the available choices by including helpful details, like using repetitive sequences or the need of cloning a gene fused with another (e.g. in reporter constructs). Yet, we consider the freedom of choice as one of the key educational instruments in this setup. We have inserted our challenges as figures to reduce the chances that these are easily found by search engines. The challenge in text form is available on request from the authors.

## Challenge 1

The 1^st^ task for the students is to properly name the protein. We originally called the gene FART (Fa‐Resonating Protein), but finding a wicked name is part of the challenge. The next step is to identify the gene coding for the FART in the *Fafarafi's* genome. How do you identify and isolate a gene that generates sounds? Once the gene has been identified, it can be amplified from genomic DNA (gDNA) if it is intronless; otherwise, it should be amplified from mRNA. Next, the isolated coding DNA fragment needs to be cloned into a suitable expression vector. The coding sequence also needs to be validated to exclude PCR‐induced mutations. The cloned gene sequence may also need to be engineered to introduce the hosts effective mRNA termination and polyadenylation (pA) sequences. Last but not least, the codon usage may need to be optimised for bacterial, yeast, human, rodent cells or other earthly host cells, to express the Jovian protein effectively.

Other important aspects relate to protein translation and folding. We share the important information that *Fafarafi* species thrive at low temperatures. Thus, the selection of suitable host organisms to express *Fafarafi* proteins is essential, as they may suffer from misfolding at higher temperatures. For example, the use of plant cells may be considered, which can effectively produce proteins at ambient temperatures (16–25°C). Other options, with relatively low or ambient operating temperatures, are frog, plant or insect cells.

We further give the hint that Fa proteins may require co‐factors (ATP, FADH, NADH or NADPH) to supply energy. This may require expression of the recombinant protein in matching Jovian cell lines. This approach may also guarantee the correct distribution of the recombinant protein in organelles or on membranes. Unfortunately, no such cell lines nor cell culture conditions have been established so far—yet another potential task for our students.

The next challenge is to create suitable instruments to detect the protein and further use it for a selected application (see below). In practice, one should consider simple systems based on a miniature microphone that detects the source and direction of sound. The sound waves generated by the Fa protein may guide isolation of “sound‐positive” cells—similar to FACS—to inspire a SACS (Sound‐Activated Cell Sorter). Sound waves could also be used to visualise “sound maps” of Petri dishes, thus indicating positive colonies.

Once the recombinant protein expression is firmly established, the *FART* gene can now be modified to generate different musical notes. When the protein structure of FART is eventually known, 3D *in silico* modelling of the protein structure may be possible followed by site‐directed mutagenesis. Mutants that display clear and reproducible changes in sound could be further mutated, to yield stable, or oscillatory, and robust signals. The students must bear in mind that many of the potential uses of such tintinnabulating protein(s) would likely be in mammalian cells or in model organisms that thrive at higher temperatures than the original organism.

Usually, students suggest a preclinical application, such as using recombinant FART‐antibody fusions to detect “tumour‐specific” antigens on neoplastic cells or to detect protein–protein interactions in cells, by applying miniature microphones to generate novel microphone‐scopes (or phonoscopes) that can locate vibrations with high resolution. Other students suggest using the FART for exploring translation, secretion or protein degradation, or for research on cell–cell communication. Our understanding of these biological processes has indeed been revolutionised by the emergence of fluorescent proteins like GFP and its many derivates. Very recently, and to our own surprise, some of our students found an article describing a protein complex that indeed produces sound. This protein complex is a gas‐filled nanostructure, used by photosynthetic organisms such as *Bacillus megaterium* and *Aphanizomenon flos‐aquae* to regulate buoyancy in their habitat 8. Upon ultrasound stimulation, these nanostructures, which have been named ARGs (acoustic reporter genes), burst releasing the gas and producing a sound. Since the size and protein structure of the ARGs collapse at different acoustic pressures, these properties would allow differential detection in practice. The nano‐flatulencies produced by sonicated ARGs have been used to localise ARG‐expressing *E. coli* and *Salmonella typhimurium* in the gastrointestinal tract, and in tumours of mice, using non‐invasive ultrasound detection 7. Suddenly, our imaginary challenge turned to be far less imaginary.

## Challenge 2

This challenge was created around the need to analyse the sequence of the alien DNA fragment and to understand the functions of its elements. As a first step, students need to clearly distinguish between coding DNA regions and regulatory ones. Here, we typically point out that this largely relies on the human/eukaryotic genetic system of the host *Homo sapiens*. The DNA fragment is flanked by inverted repeats similar to those of retroviruses, in which they may function as promoters, or as guiding sequence for genome insertion and excision. To the right of the *Ssm6* element, there is a pA site. The left part of the sequence element codes for 5 genetic elements, each separated by identical repetitive spacer sequences (TGGCACGCCACCGCC). On the right side, a second expressed gene with a pA stretch can be identified which is expressed in the opposite direction, indicated by its inverted sequence. This gene, termed “WHATAse”, is not homologous to any known gene or protein in current genome databases. The clue for understanding its putative function is conveniently provided by its name: WHATA indicates, in single‐letter amino acid code, the short peptide sequence Trp‐His‐Ala‐Thr‐Ala. The ending “Ase” indicates that it may be a protease. Intriguingly, the sequence of the 15nt repeated sequence element that separates each of the 5 genetic elements on the left is TGG‐CAC‐GCC‐ACC‐GCC. A simple comparison with the genetic code shows that this sequence codes for the five amino acids Trp‐His‐Ala‐Thr‐ Ala, which corresponds to WHATA in one‐letter amino acid code.

One of the advantages of our “designed” challenges is that each step can be questioned and interfered with. The next task is to validate the functions of the putative WHATAse. We suggest the development of a bioassay to confirm the recognition site(s). A convenient method is the use of synthetic peptides that centrally contain the WHATA element, flanked by a fluorescent dye and a quencher.

**Figure 3:**
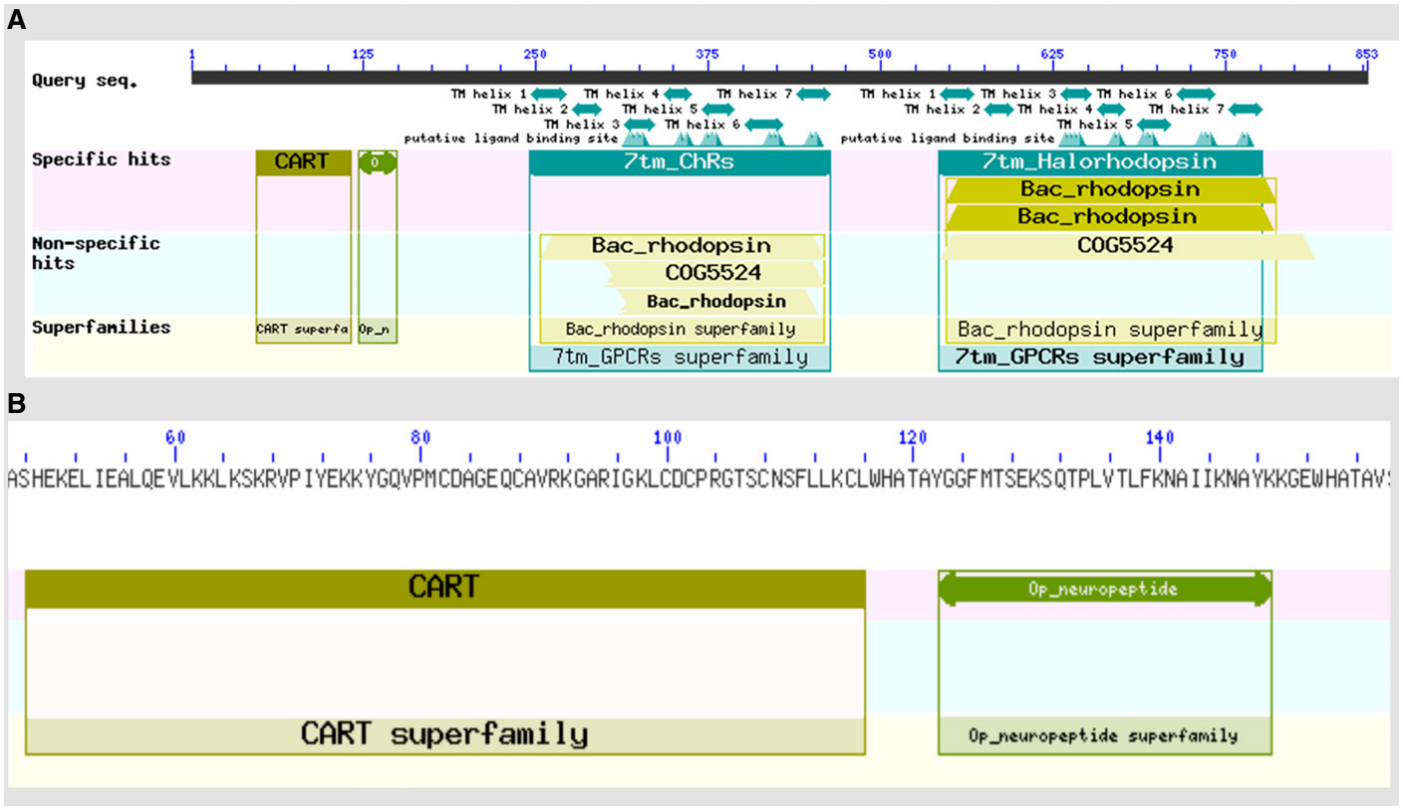
Sequence analysis of Challenge 2 Results from BLASTP alignment, showing 4 conserved domains: (A) CART sup, Op_n, 7tm‐ChRs and 7tm_Halorhodopsin. The last fragment is not displayed due to its small size. The 2nd domain (Op_n) is rather unclear and might easily be missed. (B) A closer look, at the residual level, at the first two elements (CART superfamily and Op_neuropeptide superfamily). Two WHATA sites flanking the coding sequences are visible.

For the analysis of the first genetic element, we provide the students with the full DNA sequence. We ask them to analyse this sequence as both DNA/RNA and amino acid sequences. The sequences are then compared to known genes/proteins, using BLAST (https://blast.ncbi.nlm.nih.gov/Blast.cgi). In our specific case, the BLAST sequence analysis will only result in 3 or 4 results (Fig 3). This is because two of the sequence elements are very short and masked by the rest, and no strong homologies are found in this manner. These sequences need to be analysed separately to clarify their identities.

The assumed identity and possible function of the five products (peptides and proteins) can be inferred from their level of homology to known genes. Analyses of DNA sequences typically result in weaker homologies, since the genetic code is degenerated. In contrast, BLAST analysis of protein sequences (BLASTP) can yield very high levels of homology to known proteins. The first fragment is a cocaine‐ and amphetamine‐regulated transcript, or CART. The second fragment is similar to a human β‐endorphin. Both peptides are neuropeptides and involved in the sensation of pleasure, pain and highly active in the “rewarding centre” of the human brain. It is not difficult to understand why aliens use them to control and tranquilise humans.

The next two proteins are highly similar to opsin channels. These are light‐activatable transmembrane proteins capable to polarise or depolarise neurons, provided light with the correct wavelength is used. The first peptide is highly similar to the red‐light‐activatable Na channel, ReaChR. The second protein is homologous to the halorhodopsin (NpHR) pump. This membrane pump is specific for chloride ions (Cl^−^) upon yellow light activation. Cl^−^ pumping inside the cell has the potential to inhibit the action potential in neurons.

The final segment of the inserted DNA encodes a peptide called “Ssm6”. BLAST sequence alignments based on DNA will not provide any results, whilst BLASTP results in approximately ten hits, all homologous to scoloptoxin. The students can now see that the small gene Ssm6 codes for Ssm6a, a peptide component in the venom of the Chinese red‐head centipede *Scolopendra mutilans* 9. This peptide exerts strong analgesic effects that are even more potent than morphine, but without the negative side effects of many opiates.

Finally, we come to the most critical point: saving mankind from an alien invasion, and global mind control. This challenge was originally devised to be solved by an evolved CRE recombinase, based on the excision of the HIV genome from infected cells 10. But there are many other ways to solve this challenge. Only a few years ago, gene silencing (siRNAs) or zinc finger nucleases (ZFNs) would be used, later on TALENs. This is now largely replaced by CRISPR/Cas9. A very good example to show that possible solutions to our challenges will therefore evolve as fast as science and technology does. We remind students that there are currently no working technologies, to specifically excise the inserted alien DNA in all neurons.

**Figure 4:**
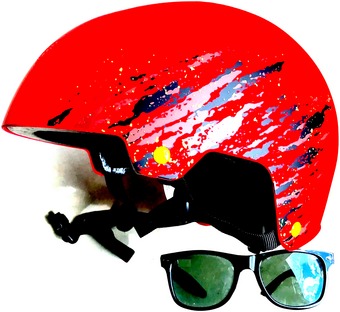
Alien radiation protection kit Possibly, one of the most interesting solutions provided by a student is shown. To avoid stimulation by laser beams, it is advised to wear an alien protection kit. This has little to do with molecular mechanism; nevertheless—we did praise this student's solution for its originality—she had a full and excellent molecular solution too. Sometimes, the most effective way to solve a difficult problem is by the simplest of ways. Image has been recreated by the authors with help (see Acknowledgements).

A more feasible and convenient way to counteract the alien infection is via pharmacological agents. High‐throughput drug testing with the goal to identify specific inhibitors against WHATAse is hinted in the challenge text. A reliable bioassay for screening purposes would provide a fast, fluorescence or luminescence‐based readout for WHATAse activity, something already mentioned above. Millions of existing small‐molecule compounds contained in chemical libraries could be tested, until an effective inhibitor is found. Ideally, it would also effectively penetrate the blood–brain barrier. One of our students devised an ingenious way to protect the *brain barrier* instead (Fig 4).

These challenges have been inspired by real science and follow a logic similar to real‐life scientific projects. They always involve multiple steps and the use of different tools. As with empirical science, they are based on imagination, generating scientific hypotheses, and testing.

## Conclusions

There is a need to teach young scientists that good science means that biomedical phenomena must be thoroughly studied, often using different angles and techniques, and ideally avoiding personally biased views or preferences or prejudices by others. After all, when a distinguished scientist states that something is impossible, he is very probably wrong. However, there are only a handful of really challenging, thought‐provoking teaching programmes for students, such as the R3 programme^C^. There seems to be resistance even from teachers towards these more challenging projects, as these require additional work, more preparation and thorough evaluation. Another problem is that academic institutions often want proof by standardised examinations, which are part of the official curriculum. In our case, we solved this problem by inviting a panel of colleagues to evaluate the students. One thing we can assure: enthusiastic and promising students will always be noticed.

Students deserve an education that prepares them for the critical moment when they start actual scientific research. Currently, they are often overloaded with disconnected information, which comes mainly as “facts”. But what may matter most is often skipped—the questioning, philosophising, planning, testing and constantly going back to the drawing board. To make teaching more vivid, similar approaches have been successfully introduced in the biomedical sciences 2, such as the “flipped classroom” concept that also drags students out of their usual comfort zone—which in practice is mainly a zone of convenient inactivity and boredom. Somewhat comparable “critical thinking exercises” (a.k.a role plays or simulations) have been introduced in engineering—with the goal of confronting students with real‐life examples that prepares them for the demands of the modern day workplace (https://www.sefi.be/wp-content/uploads/2017/09/56744-G.-KLADIS.pdf). Solving difficult challenges is clearly not just meant to pass an examination. It is mainly about retracing the steps to the very reasons we became scientists in the first place. Let us try to bring the excitement back to teaching.

## Conflict of interest

The authors declare that they have no conflict of interest.
